# Isolation and molecular characterization of multidrug-resistant *Escherichia coli* from chicken meat

**DOI:** 10.1038/s41598-020-78367-2

**Published:** 2020-12-15

**Authors:** Md. Masudur Rahman, Asmaul Husna, Hatem A. Elshabrawy, Jahangir Alam, Nurjahan Yasmin Runa, A. T. M. Badruzzaman, Nahid Arjuman Banu, Mohammad Al Mamun, Bashudeb Paul, Shobhan Das, Md. Mahfujur Rahman, A. T. M. Mahbub-E-Elahi, Ahmed S. Khairalla, Hossam M. Ashour

**Affiliations:** 1 Department of Pathology, Faculty of Veterinary, Animal and Biomedical Sciences, Sylhet Agricultural University, Sylhet, 3100 Bangladesh; 2 ABEx Bio-Research Center, Dhaka, Bangladesh; 3 Department of Molecular and Cellular Biology, College of Osteopathic Medicine, Sam Houston State University, Conroe, TX 77304 USA; 4 Animal Biotechnology Division, National Institute of Biotechnology, Savar, Dhaka 1349 Bangladesh; 5 Department of Livestock Services, Farmgate, Dhaka 1215 Bangladesh; 6 Department of Anatomy and Histology, Faculty of Veterinary, Animal and Biomedical Sciences, Sylhet Agricultural University, Sylhet, 3100 Bangladesh; 7 Department of Medicine, Faculty of Veterinary, Animal and Biomedical Sciences, Sylhet Agricultural University, Sylhet, 3100 Bangladesh; 8 Department of Microbiology and Immunology, Faculty of Veterinary, Animal and Biomedical Sciences, Sylhet Agricultural University, Sylhet, 3100 Bangladesh; 9 Microbiology and Immunology Department, Faculty of Pharmacy, Beni-Suef University, Beni-Suef, 62514 Egypt; 10 Department of Biology, University of Regina, Saskatchewan, Canada; 11 Department of Integrative Biology, College of Arts and Sciences, University of South Florida, St. Petersburg, FL 33701 USA; 12 Department of Microbiology and Immunology, Faculty of Pharmacy, Cairo University, Cairo, 11562 Egypt

**Keywords:** Antimicrobials, Bacteria

## Abstract

Antibiotic-resistant *Escherichia coli* (*E. coli*) are common in retail poultry products. In this study, we aimed to isolate and characterize multidrug resistant (MDR) *E. coli* in raw chicken meat samples collected from poultry shops in Sylhet division, Bangladesh, as well as to determine correlation between resistance phenotype and genotype. A total of 600 chicken meat swabs (divided equally between broiler and layer farms, *n* = 300 each) were collected and the isolates identified as *E. coli* (*n* = 381) were selected. Disc diffusion antimicrobial susceptibility assay showed resistance of these isolates to ampicillin, erythromycin, tetracycline, streptomycin, trimethoprim-sulfamethoxazole, chloramphenicol, and gentamicin. Polymerase chain reaction (PCR) identified several antibiotic resistance genes (ARGs) in our isolates. Among these ARGs, the prevalence of *tetA* (for tetracycline) was the highest (72.58%) in broiler chicken isolates, followed by *sul*1 (for sulfonamide; 44.16%), *aadA*1 (for streptomycin; 33.50%), *ereA* (for erythromycin; 27.41%), *aac-3-IV* (for gentamicin; 25.38%), and the two genes *cmlA* (24.87%) and *catA1* (8.63%) for chloramphenicol. On the other hand, the respective prevalence in layer chicken isolates were 82.06%, 47.83%, 35.87%, 35.33%, 23.91%, 19.02%, and 5.43%. Furthermore, 49.23% of the isolates from broiler chicken were MDR, with the presence of multiple antibiotic resistance genes, including 3 (40.11%) and 4 (9.13%) genes. On the other hand, 51.09% of layer chicken *E. coli* isolates were MDR, with 3, 4 or 5 ARGs detected in 36.41%, 14.13%, and 0.54% of the isolates, respectively. We also found that 12.8% of broiler chicken *E. coli* isolates and 7.61% of layer chicken isolates carried genes coding for extended-spectrum SHV beta-lactamases. Lastly, we report the presence of the AmpC beta-lactamase producing gene (CITM) in 4.56% and 3.26% of broiler and layer chicken *E. coli* isolates, respectively. We found significant correlations between most of the antimicrobial resistant phenotypes and genotypes observed among the investigated *E. coli* isolates. Our findings highlight the need for the prudent use of antimicrobials in chickens to minimize the development of antibiotic-resistant bacterial strains.

## Introduction

Antimicrobial resistance has recently become a public health concern. In response to the problem, the World Health Organization (WHO) has recommended a global surveillance system in veterinary and human medicine. The theme of the 2011 World Health Day was “Antibiotic resistance: no action today, no cure tomorrow” and it was selected to create mass awareness among the world population.

*Escherichia coli* (*E. coli*), a member of the Enterobacteriaceae family and a major cause of foodborne infections, is a common inhabitant of gastrointestinal tract of poultry, animals, and humans^1^. Unhygienic slaughter practices are responsible for contamination of meat with *E. coli*^2^. It has been reported that the *E. coli* strains isolated from contaminated meat and meat products are resistant to commonly used antibiotics^3^. Excessive use of antibiotics is considered the main cause of antibiotic resistance^4,5^. This resistance is acquired through horizontal gene transfer or gene mutations^6,7^. Multidrug-resistant (MDR) bacteria usually harbour several drug resistant genes^8^. The rapid emergence of multidrug-resistant *E. coli* strains has resulted in significant morbidity and mortality in humans^9^.

Beta-lactamases are bacterial enzymes which confer resistance to beta-lactam antibiotics, such as penicillin and cephalosporin by hydrolysing the beta-lactam ring. In recent years, new types of beta-lactamase enzymes including extended-spectrum beta-lactamases (ESBLs) and AmpC beta-lactamases have emerged^10–12^. The most common beta-lactamases in Gram-negative bacteria are TEM, SHV, OXA, CMY, and CTX-M beta-lactamases^13^. ESBLs and AmpCs are mostly located on mobile genetic elements (plasmids or integrons). These mobile genetic elements get transferred to other bacterial cells through horizontal gene transfer mechanisms, including conjugation, transformation, and transduction^14^. Food animals as well as retail meat act as reservoir of ESBL and AmpC-producing *E. coli*^15–18^.

Bangladesh is a large poultry producer. According to the report published in 2015 by the Department of Livestock Services, there were over 115,000 farms, producing approximately 170 million broiler and layer chickens in Bangladesh. Like many other developing countries, hygienic raw food processing technology is still underdeveloped and there is lack of proper antimicrobial drug resistance surveillance in Bangladesh. Uncontrolled use of antimicrobials for the prevention and/or treatment of diseases of food animals increases the risk of emergence of resistant bacterial strains. Contamination of chicken meat with ESBL and AmpC-producing *E. coli* is currently becoming an emerging food safety concern in Bangladesh. However, limited information is available on the prevalence and the genotypic characteristics of antibiotic-resistant bacterial strains associated with humans’ or food animals’ ecological niches in Bangladesh.

In this study, we identified and isolated *E. coli* from broiler and layer chicken meat from retail poultry shops in Sylhet division of Bangladesh. In addition, the resistance of these isolates to commonly used antibiotics, such as tetracycline and others was tested. Multiplex and uniplex polymerase chain reaction (PCR) assays were used to test for several non-beta lactam antibiotic resistance genes, such as tetracycline resistance gene (*tetA*), as well as genes involved in beta-lactam antibiotic resistance*,* ESBL genes (TEM, CTX-M, CTX-M-1, CTX-M-2, SHV), and AmpC (CITM)*.*

## Materials and methods

### Ethics statement

The handling of animals in the study was performed in accordance with the current Bangladesh legislation (Cruelty to Animals Act 1920, Act No. I of 1920 of the Government of the People’s Republic of Bangladesh). The specific experiments were approved by the Ethics Committee of Sylhet Agricultural University and National Institute of Biotechnology, Bangladesh.

### Isolation and identification of *E. coli*

A total of 600 swabs were collected randomly from broiler (*n* = 300) and layer (*n* = 300) chicken meat samples, derived from 100 different retail poultry shops at Sylhet division of Bangladesh. Gram staining, growth characteristics on culture media (including nutrient broth, nutrient agar, MacConkey’s agar, Eosin Methylene Blue agar; all from Merck, Germany), and results of biochemical tests (including sugar fermentation, indole, methyl red (MR), Voges-Proskauer (VP), and citrate utilization tests) were used for identification and isolation of *E. coli*, as previously described^19^. Molecular confirmation of the isolates was performed using PCR targeting the 16S rRNA, using a primer set specific for *E. coli*, as previously described^20^. The isolates identified as *E. coli* (*n* = 381; 197 from broiler and 184 from layer chickens) were selected for further investigation.

### Antimicrobial susceptibility testing

The susceptibilities of the 381 chicken meat-derived *E. coli* isolates to a panel of commonly used antibiotics were determined using the Kirby-Bauer method on Mueller–Hinton agar plates (Merck, Germany) according to the guidelines and breakpoints of the Clinical and Laboratory Standard Institute^21^. The antimicrobial discs used, which were all obtained from Oxoid (UK), included: trimethoprim-sulphamethoxazole (23.75 µg), chloramphenicol (30 µg), erythromycin (15 µg), gentamicin (10 µg), tetracycline (30 µg), streptomycin (10 µg), and ampicillin (10 µg). *E. coli* ATCC 25,922 (American Type Culture collection, Manassas, VA, USA) was used as a control strain. Test results were only validated when the diameters of the inhibition zones of the *E. coli* ATCC 25922 control strain were within the performance ranges. Resistant and intermediate resistant isolates were considered as non-susceptible as previously described^22^. *E. coli* was defined as multidrug resistant isolate when it was found non-susceptible to at least one agent in three or more different classes of antimicrobial agents, excluding the broad-spectrum penicillins without a β-lactamase inhibitor^22^.

### Extraction of bacterial genomic DNA

All *E. coli* isolates (*n* = 381) were cultured overnight in nutrient broth at 37 °C and then bacterial genomic DNA was extracted using the Phenol–Chloroform Isoamyl Alcohol (PCI) method, as described previously^23^. The average concentration and purity of the extracted DNA were determined using the Nano Drop™ 2000c spectrophotometer (ThermoScientific, USA).

### PCR amplification and detection of antibiotic resistant genes

All the tested *E. coli isolates* (*n* = 381) were PCR-screened for the presence of seven non-beta-lactam and six beta-lactam antibiotic resistant genes (ARGs) using a combination of two uniplex and two multiplex assays. The resistant genes for tetracycline (*tetA*) and streptomycin (*aadA1*) were amplified individually using set 1 and 2 primers, respectively (Table 1). Set 3 and 4 primers were used to detect some other ARGs (*sul1*, *catA1*, *cmlA*, *ereA*, *aac-3-IV, bla*SHV, and CITM) and four types of ESBL genes (*bla*TEM, *bla*CTX-M, *bla*CTX-M-1, and *bla*CTX-M-2), respectively (Table 1). All PCR amplifications were conducted in a thermal cycler (Gene Atlas, Japan) using the conditions listed below. The basic setup of the uniplex PCR amplification for set 1 consisted of an initial denaturation step at 95 °C for 15 min, followed by denaturation at 94 °C for 30 s, annealing at 56 °C for 30 s, and extension at 72 °C for 1 min. This cycle was repeated 30 times followed by a final extension step at 72 °C for 10 min^24^. The uniplex PCR amplification conditions for set 2 consisted of an initial denaturation step at 95 °C for 3 min, followed by denaturation at 94 °C for 1 min, annealing at 58 °C for 90 s, and extension at 72 °C for 1 min. This cycle was repeated 35 times followed by a final extension step at 72 °C for 10 min^25^. Regarding the basic setup of the multiplex PCR for primer set 3, it consisted of an initial denaturation step at 95 °C for 15 min, followed by denaturation at 94 °C for 1 min, annealing at 58 °C for 30 s, and extension at 72 °C for 1 min. This cycle was repeated 30 times followed by a final extension step at 72 °C for 10 min^24^. Finally, the thermal profile of the multiplex PCR with primer set 4 included an initial denaturation step at 94 °C for 3 min, followed by denaturation at 94 °C for 1 min, annealing at 55 °C for 1 min, and extension at 72 °C for 1 min. This cycle was repeated 30 times followed by a final extension step at 72 °C for 10 min^26,27^. After amplification, 10 µl of each PCR reaction was separated on a 1.5% (w/v) agarose gel electrophoresis using QA-AgaroseTM (MP Biomedical, USA), stained with ethidium bromide (0.5 mg/ml), and visualized using a gel documentation system (Uvitech, UK). A molecular weight marker with 100 bp increments (100 bp DNA ladder, Invitrogen™, Massachusetts, USA) was used as a size standard. Strains of *E. coli* O157:K88ac:H19, CAPM 5933 and *E. coli* O159:H20, CAPM 6006 were used as positive controls, while distilled water was used as a negative control.

**Table 1:** List of primers used in the current study for uniplex (sets 1 and 2) and multiplex (sets 3 and 4) PCR assay formats for the amplification of beta-lactam and non-beta-lactam ARGs in the investigated *E. coli* isolates.

Set	Antibacterial agent	Target gene	Primer	Primer sequence (5′ → 3′ direction)	Amplicon size (bp)	Annealing temperature (°C)	References
1	Tetracycline	*tetA*	*tetA*-F	GGT TCA CTC GAA CGA CGT CA	577	56	^28^
			*tetA*-R	CTG TCC GAC AAG TTG CAT GA			
2	Streptomycin	*aadA1*	*aadA1*-F	TAT CCA GCT AAG CGC GAA CT	447	58	^24^
			*aadA1*-R	ATT TGC CGA CTA CCT TGG TC			
3	Sulfonamide	*sul1*	*sul1*-F	TTC GGC ATT CTG AAT CTC AC	822	58	^24^
			*sul1*-R	ATG ATC TAA CCC TCG GTC TC			
	Erythromycin	*ereA*	*ereA*-F	GCC GGT GCT CAT GAA CTT GAG	419	58	^24^
			*ereA*-R	CGA CTC TAT TCG ATC AGA GGC			
	Chloramphenicol	*cmlA*	*cmlA*-F	CCG CCA CGG TGT TGTTGT TAT C	698	58	^24^
			*cmlA*-R	CAC CTT GCC TGC CCA TCA TTA G			
	Chloramphenicol	*catA1*	*catA1*-F	AGT TGC TCA ATG TAC CTA TAA CC	547	58	^24^
			*catA1*-R	TTG TAA TTC ATT AAG CAT TCT GCC			
	Gentamicin	*aac-3-IV*	*aac-3-IV*-F	CTT CAG GAT GGC AAG TTG GT	286	58	^24^
			*aac-3-IV*-R	TCA TCT CGT TCT CCG CTC AT			
	AmpC’s	CITM	CITM-F	TGG CCA GAA CTG ACA GGC AAA	462	58	^24^
			CITM-R	TTT CTC CTG AAC GTG GCT GGC			
	Beta-lactam	*bla*SHV	SHV-F	TCG CCT GTG TAT TAT CTC CC	768	58	^24^
			SHV-R	CGC AGA TAA ATC ACC ACA ATG			
4	Beta-lactam	*bla*TEM	TEM-F	GCG GAA CCC CTA TTT G	964	55	^29^
			TEM-R	ACC AAT GCT TAA TCA GTG AG			
	Beta-lactam	*bla*CTX-M	CTX-M-F	ATG TGC AGY ACC AGT AAR GTK ATG GC	592	55	^30^
			CTX-M-R	TGG GTR AAR TAR GTS ACC AGA AYS AGC GG			
	Beta-lactam	*bla*CTX-M-1	CTX-M-1-F	GGT TAA AAA ATC ACT GCG TC	863	55	^31^
			CTX-M-1-R	TTG GTG ACG ATT TTA GCC GC			
	Beta-lactam	*bla*CTX-M-2	CTX-M-2-F	GAT GAG ACC TTC CGT CTG GA	397	55	^26^
			CTX-M-2-R	CAG AAA CCG TGG GTT ACG AT			

*F* forward primer, *R* reverse primer.

### Statistical analysis

The antibiotic resistance data are expressed as percentages or frequency of the *E. coli* isolates. A two-way analysis of variance (ANOVA) without replication was used to determine the significant differences in the levels of resistance prevalence among the selected antibiotics, between broiler and layer chickens, as well as among the four districts under study. A *P* value of < 0.05 was considered to be statistically significant. These statistical analyses were carried out using the GraphPad Prism (version 6; GraphPad Software Inc.; USA).

## Results

### Prevalence of *E. coli* in broiler and layer chicken meat swabs

We collected a total of 600 chicken meat swab samples (75 from broiler and 75 from layer chickens from each of the four districts of Sylhet division; Sylhet, Moulavibazar, Sunamganj, and Habiganj). Out of the 600 samples, 381 *E. coli* isolates (63.5%) (197 from broiler and 184 from layer chicken) were identified using staining, cultural, and biochemical tests (Table 2). There was no statistically significant difference in the prevalence of *E. coli* between samples from broiler and layer chickens (*P* = 0.06), nor among the four districts (*P* = 0.37).

**Table 2:** Prevalence of *E. coli* in broiler or layer chicken meat swab specimens, collected from various retail shops at Sylhet division.

No. (%) of *E. coli* isolates						
Sample	Sylhet (*n* = 150)	Moulavibazar (*n* = 150)	Sunamganj (*n* = 150)	Habiganj (*n* = 150)	Total (*n* = 600)	*P*-value^a^
Broiler (*n* = 300)	48 (64%)	48 (64%)	49 (65.33%)	52 (69.33%)	197 (65.67%)	0.06^#^
Layer (*n* = 300)	46 (61.33%)	44 (58.67%)	48 (64%)	46 (61.33)	184 (61.3%)	0.37^##^
Total (*n* = 600)	94 (62.67%)	92 (61.33%)	97 (64.67%)	98 (65.33%)	381 (63.5%)	

^*a*^*P *values were calculated using a two-way analysis of variance (ANOVA) without replication. *P* values > 0.05 were considered to be statistically nonsignificant.

^#^Variance between broiler and layer chickens.

^##^Variance among the four districts under study.

### Prevalence of antimicrobial resistance

All *E. coli* isolates (*n* = 381) were tested for resistance to seven different antimicrobial agents by disc diffusion method. As shown in Table 3, 286 (75.06%) of the isolates were MDR. Resistance to ampicillin, erythromycin, and tetracycline were the most prevalent in the isolates (98.95%, 89.5%, and 85.3%, respectively). No significant difference in resistance patterns was observed in isolates from broiler and layer chicken meat (*P* = 0.18).

**Table 3:** Antimicrobial resistance rates among the investigated *E. coli* (*n* = 381) isolates in relation to type of hens (broiler or layer) in Sylhet division of Bangladesh.

Antimicrobial agents	No. (%) of *E. coli* isolates phenotypically resistant to			Total (*n* = 381)
	Broiler (*n* = 197)	Layer (*n* = 184)	*P*-value^a^	
Trimethoprim-Sulfamethoxazole	101 (51.26%)	106 (57.60%)	0.18^#^	207 (54.33%)
Chloramphenicol	105 (53.29%)	85 (46.19%)	**0.0003**^##^	190 (49.86%)
Erythromycin	164 (83.24%)	177 (96.19%)		341 (89.50%)
Gentamicin	55 (27.91%)	50 (27.17%)		105 (27.55%)
Tetracycline	160 (81.21%)	165 (89.67%)		325 (85.30%)
Streptomycin	120 (60.91%)	150 (81.52%)		270 (70.86%)
Ampicillin	197 (100%)	180 (97.82%)		377 (98.95%)
Multidrug resistant (≥ 3 antibiotics)	154 (78.17%)	132 (71.73%)		286 (75.06%)

^a^*P *values were calculated using a two-way analysis of variance (ANOVA) without replication. *P* values < 0.05 are highlighted in bold.

^#^Variance between broiler and layer chickens.

^##^Variance among the antimicrobial agents.

### Prevalence of non-beta-lactam ARGs

The overall prevalence of non-beta-lactam ARGs among the investigated *E. coli* isolates in relation to type of hens (broiler or layer) in Sylhet division are given in Table 4. The most prevalent gene was that of *tetA* (for tetracycline resistance; harboured by 77.17% of the isolates), which was followed by *sul1* (for sulphonamide resistance; 45.94%), *aadA1* (for streptomycin resistance; 34.65%), *ereA* (for erythromycin resistance; 31.23%), *aac-3-IV* (for gentamicin resistance; 24.67%), and the two genes *cmlA* (22.05%) and *catA1* (7.09%) for chloramphenicol resistance. Additionally, there was a significant difference in the prevalence of the seven non-beta-lactam ARGs among the *E. coli* isolates (*P* = 0.0001) but there was no significant difference in the prevalence of each antibiotic resistance gene in broiler versus layer chickens (*P* = 0.42).

**Table 4:** Overall prevalence of non-beta-lactam ARGs among the investigated *E. coli* (*n* = 381) isolates in relation to type of hens (broiler or layer) in Sylhet division of Bangladesh.

No. (%) of *E. coli* isolates								
Sample	*sul1*	*cmlA*	*catA1*	*ereA*	*aac-3-IV*	*tetA*	*aadA1*	*P*-value^*a*^
Broiler (*n* = 197)	87 (44.16%)	49 (24.87%)	17 (8.63%)	54 (27.41%)	50 (25.38%)	143 (72.58%)	66 (33.5%)	0.42^#^
Layer (*n* = 184)	88 (47.83%)	35 (19.02%)	10 (5.43%)	65 (35.33%)	44 (23.91%)	151 (82.06%)	66 (35.87%)	**0.0001**^##^
Total (*n* = 381)	175 (45.94%)	84 (22.05%)	27 (7.09%)	119 (31.23%)	94 (24.67%)	294 (77.17%)	132 (34.65%)	

^a^*P *values were calculated using a two-way analysis of variance (ANOVA) without replication. *P* values < 0.05 are highlighted in bold.

^#^Variance between broiler and layer chickens.

^##^Variance among the antibiotic resistant genes.

As mentioned previously, the investigated *E. coli* isolates were from broiler and layer chickens that have been collected from four districts within Sylhet division. The prevalence of *E. coli* isolates from broiler or layer chicken harbouring non-beta-lactam resistance genes in relation to these districts is shown in Table 5. There was no significant difference in the prevalence of *sul1, catA1, ereA, aac-3-IV, tetA* and *aadA1*genes between broiler and layer chickens, nor between the four districts (*P* > 0.05). On the other hand, the prevalence of *cmlA* gene was significantly higher in broiler chicken than in layer chicken. Moreover, the prevalence of *cmlA* gene was significantly different among the four districts under study (*P* < 0.05).

**Table 5:** Prevalence and distribution of non-beta-lactam ARGs among the investigated *E. coli* (*n* = 381) isolates in relation to the four districts of Sylhet division under study.

No. (%) of *E. coli* isolates					
Sample	Sylhet	Moulavibazar	Sunamganj	Habiganj	*P*-value^*a*^
***sul1 (sulfonamide resistance gene)***					
Broiler	21 (43.75%)	22 (45.83%)	23 (46.94%)	21 (40.38%)	0.16^#^
Layer	21 (46.65%)	24 (54.55%)	25 (52.08%)	18 (39.13%)	0.1^##^
***catA1 (chloramphenicol resistance gene)***					
Broiler	5 (10.42%)	4 (8.33%)	4 (8.16%)	4 (7.69%)	0.11^#^
Layer	3 (6.52%)	1 (2.27%)	2 (4.17%)	4 (8.69%)	0.48^##^
***cmlA (chloramphenicol resistance gene)***					
Broiler	12 (25%)	14 (29.17%)	11 (22.45%)	12 (23.07%)	**0.0001**^#^
Layer	9 (19.57%)	10 (22.73%)	8 (16.67%)	8 (17.39%)	**0.0007**^##^
*ereA* (erythromycin resistance gene)					
Broiler	13 (27.08%)	12 (23.07%)	14 (28.57%)	14 (26.92%)	0.21^#^
Layer	19 (41.31%)	20 (45.45%)	12 (25%)	14 (30.43%)	0.73^##^
***aac-3-IV (gentamicin resistance gene)***					
Broiler	12 (25%)	14 (29.17%)	13 (26.53%)	11 (21.15%)	0.39^#^
Layer	10 (21.73%)	11 (25%)	12 (25%)	11 (23.91%)	0.31^##^
***tetA (tetracycline resistance gene)***					
Broiler	35 (72.91%)	34 (70.83%)	35 (71.43%)	39 (75%)	0.27^#^
Layer	40 (86.96%)	33 (65%)	42 (87.5%)	36 (78.26%)	0.44^##^
***aadA1 (streptomycin resistance gene)***					
Broiler	16 (33.33%)	17 (35.42%)	17 (34.69%)	16 (30.78%)	0.36^#^
Layer	17 (36.96%)	14 (31.81%)	18 (37.5%)	17 (36.96%)	0.82^##^

^a^*P *values were calculated using a two-way analysis of variance (ANOVA) without replication. *P* values < 0.05 are highlighted in bold.

^#^Variance between broiler and layer chickens.

^##^Variance among the four districts under study.

### Prevalence of MDR genes

**Figure 1:**
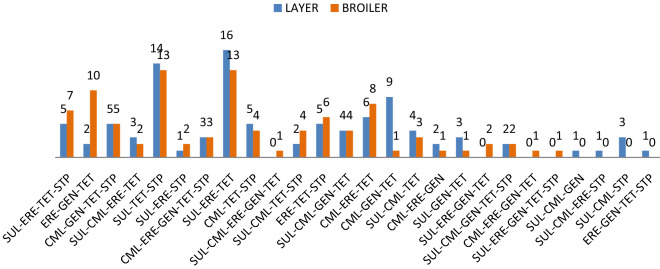
MDR profiles of *E. coli* isolates from broiler (*n* = 97) and layer (*n* = 94) chickens. *SUL* sulfonamide (a representative of folate pathway inhibitors), *CML* chloramphenicol (a representative of phenicols), *ERE* erythromycin (a representative of macrolides), *TET* tetracycline (a representative of tetracyclines), *GEN* gentamicin, *STP* streptomycin (representatives of aminoglycosides).

MDR analysis was carried out according to the definition proposed previously^22^. The analysis was performed against five antimicrobial categories (representative antimicrobials tested in this analysis and the respective genes involved are shown in brackets): aminoglycosides (gentamicin-*aac-3-IV,* streptomycin-*aadA1*), tetracyclines (tetracycline-*tetA*), phenicols (choloramphenicol-*cmlA and catA1*), macrolides (erythromycin-*ereA*) and folate pathway inhibitors (sulfonamide/trimethoprim-*sul1*). The analysis showed 26 resistance profiles (Fig. 1), the most frequent among which (*n* = 16) correlated with isolates from layer chickens and harboured the resistance genes for sulfonamide, erythromycin and tetracycline.

A total of 191 (50.13%) out of 381 *E. coli* isolates from broiler and layer chickens of Sylhet division carried more than 3 ARGs in their genomes. The majority of the *E. coli* isolates harboured resistance genes to three classes of antibiotics (146 isolates; 38.32%), while 44 isolates (11.55%) and 1 isolate (0.26%) possessed four and five antibiotic resistance genes, respectively (Table 6). Within the triple-antibiotic resistant *E. coli* isolates, 79 isolates were from broiler chickens, while 67 isolates were from layer chickens. Out of the 44 isolates that carried four antibiotic resistance genes, 18 (9.13%) were from broiler chickens and 26 (14.13%) were from layer chickens. However, in contrast to the one *E. coli* layer chicken isolate (0.54%), none of the broiler chicken isolates carried five antibiotic resistance genes. There was a significant difference in the prevalence of MDR genes (*P* = 0.01); however, there was no significant difference in their prevalence between broiler and layer chickens (*P* = 0.83).

**Table 6:** Overall prevalence of MDR genes among the investigated *E. coli* (*n* = 381) isolates in relation to type of hens (broiler or layer) in Sylhet division of Bangladesh.

*E. coli* isolates	No. (%) of *E. coli* carrying MDR genes			Total MDR	*P*-value^a^
	3 genes	4 genes	5 genes		
Broiler (*n* = 197)	79 (40.11%)	18 (9.13%)	0 (0%)	97 (49.23%)	0.83^#^
Layer (*n* = 184)	67 (36.41%)	26 (14.13%	1 (0.54%)	94 (51.09%)	**0.01**^##^
Total (*n* = 381)	146 (38.32%)	44 (11.55%)	1 (0.26%)	191(50.13%)	

^a^*P *values calculated using a two-way analysis of variance (ANOVA) without replication. *P* values < 0.05 are highlighted in bold.

^#^Variance between broiler and layer chickens.

^##^Variance among the classes of MDR genes.

Next, we aimed to determine the prevalence of *E. coli* isolates (from broiler or layer chicken) harbouring MDR genes (non-beta lactam antibiotics) in relation to the four districts of Sylhet division under study (Table 7). Within the three categories of MDR isolates (those having 3, 4, or 5 MDR genes), all the prevalence differences between the four districts were found to be non-significant (*P* > 0.05). Similarly, there were no significant differences in the prevalence of MDR isolates between broiler and layer chickens, with the exception of isolates with 4 MDR genes. For this category, we observed a significant difference (*P* = 0.01) in the prevalence of MDR isolates between the two types of hens.

**Table 7:** Prevalence and distribution of MDR genes among the investigated *E. coli* (*n* = 381) isolates in relation to the four districts of Sylhet division under study.

No. (%) of *E. coli* isolates					
Sample	Sylhet	Moulavibazar	Sunamganj	Habiganj	*P*-value^*a*^
***E. coli with 3 MDR genes***					
Broiler	19 (39.58%)	24 (50%)	16 (32.65%)	20 (38.46%)	0.23^#^
Layer	15 (32.61%)	21 (47.73%)	17 (35.42%)	14 (30.43%)	0.06^##^
***E. coli with 4 MDR genes***					
Broiler	5 (10.42%)	4 (8.33%)	5 (10.2%)	4 (7.69%)	**0.01**^#^
Layer	7 (15.22%)	7 (15.91%)	6 (12.5%)	6 (13.04%)	0.51^##^
***E. coli with 5 MDR genes***					
Broiler	0 (0%)	0 (0%)	0 (0%)	0 (0%)	0.39^#^
Layer	1(2.17%)	0 (0%)	0 (0%)	0 (0%)	0.5^##^

^a^*P *values were calculated using a two-way analysis of variance (ANOVA) without replication. *P* values < 0.05 are highlighted in bold.

^#^Variance between broiler and layer chickens.

^##^Variance among the four districts under study.

### Detection of beta-lactamase coding genes (ESBL and AmpC)

Out of 381 *E. coli* isolates, 53 (13.91%) harboured beta-lactam ARGs in their genomes. Additionally, 38 isolates (10%) were positive for the SHV ESBL gene, whereas 15 (3.93%) were positive for AmpC (CITM) gene (Table 8). Out of 197 resistant *E. coli* isolates from broiler chicken, 24 (12.18%) and 9 (4.56%) were positive for SHV and CITM genes, respectively. Similarly, out of 184 *E. coli* isolates from layer chicken, 14 (7.61%) and 6 (3.26%) were positive for SHV and CITM genes, respectively. None of the investigated *E. coli* isolates (whether from broiler or layer chickens) contained TEM, CTX-M, CTX-M-1, or CTX-M-2 genes. Therefore, in total, 16.75% (*n* = 33) of broiler chicken *E. coli* isolates carried either ESBL or AmpC genes, compared to 10.87% (*n* = 20) of layer chicken *E. coli* isolates. Statistically significant differences between the prevalence of both types of beta-lactamase coding genes (ESBL and AmpC) were observed (*P* = 0.001) but there were no significant differences between broiler and layer chickens (*P* = 0.24).

**Table 8:** Overall prevalence of beta-lactam (ESBL and AmpC) antibiotic resistant genes among the investigated *E. coli* (*n* = 381) isolates in relation to type of hens (broiler or layer) in Sylhet division of Bangladesh.

No. (%) of *E. coli* isolates								
Sample	Selected ESBL genes					AmpC gene	Total (ESBL + AmpC)	*P*-value^*a*^
	TEM	CTX-M	CTX-M-1	CTX-M-2	SHV	CITM		
Broiler (*n* = 197)	0 (0%)	0 (0%)	0 (0%)	0 (0%)	24 (12.18%)	9 (4.56%)	33 (16.75%)	0.24^#^
Layer (*n* = 184)	0 (0%)	0 (0%)	0 (0%)	0 (0%)	14 (7.61%)	6 (3.26%)	20 (10.87%)	**0.001**^##^
Total (*n* = 381)	0 (0%)	0 (0%)	0 (0%)	0 (0%)	38 (10%)	15 (3.93%)	53 (13.91%)	

^a^*P *values calculated using a two-way analysis of variance (ANOVA) without replication. *P* values < 0.05 are highlighted in bold.

^#^Variance between broiler and layer chickens.

^##^Variance among the beta-lactam antibiotic resistant genes.

The data regarding the prevalence of beta-lactam (ESBL and AmpC) ARGs among the investigated isolates (from broiler or layer chickens) in relation to the four districts of Sylhet division are shown in Table 9. There was a statistically significant higher prevalence of isolates possessing the SHV (ESBL) gene in broiler chicken than in layer chicken (*P* = 0.01), while there were no significant differences among the four districts (*P* = 0.07). In the case of the CITM gene (AmpC), there was no significant differences in the prevalence of isolates possessing this gene between broiler and layer chickens, nor between the four districts (*P* > 0.05).

**Table 9:** Prevalence and distribution of beta-lactam (ESBL and AmpC) antibiotic-resistant genes among the investigated *E. coli* (*n* = 381) isolates in relation to the four districts of Sylhet division under study.

Gene	Sample	Sylhet	Moulavibazar	Sunamganj	Habiganj	*P*-value^a^
SHV (ESBL)	Broiler	6 (12.5%)	7 (14.58%)	6 (12.24%)	5 (9.62%)	**0.01**^#^
	Layer	3 (6.52%)	5 (11.36%)	3 (6.25%)	3 (6.52%)	0.07^##^
CITM (AmpC)	Broiler	2 (4.55%)	4 (9.30%)	2 (4.65%)	1(2.17%)	0.41^#^
	Layer	2 (4.55%)	1 (2.56%)	2 (4.65%)	1 (2.44%)	0.57^##^

^a^*P *values calculated using a two-way analysis of variance (ANOVA) without replication. *P* values < 0.05 are highlighted in bold.

^#^Variance between broiler and layer.

^##^Variance among the four districts under study.

### Correlation between antimicrobial resistant phenotypes and genotypes

In this study, significant correlations (*r*^*2*^ > 0 and *P* < 0.05) were found between most of the antimicrobial resistant (AMR) phenotypes and genotypes observed among the investigated *E. coli* (n = 381) isolates (Table 10). Comparatively stronger correlation was found between Gentamycin and *aac-3-IV* among the *E. coli* strains isolated from layer chicken (*r*^*2*^ = 0.791 and *P* < 0.001). On the other hand, no significant correlation was observed between chloramphenicol AMR phenotype and *catA1* gene among isolates from broiler chicken (*r*^*2*^ = 0.018 and *P* = 0.067). Similarly, in the case of layer chicken, no significant correlation was observed between erythromycin AMR phenotype and *ereA* gene among the *E. coli* isolates (*r*^*2*^ = 0.001 and *P* = 0.672).

**Table 10:** Correlation between the AMR phenotypes and genotypes among the investigated *E. coli* (*n* = 381) isolates in relation to the type of hens (broiler or layer).

Type of hens	AMR^a^	Characteristics of strains					Correlation determinants	
		n-Pr^b^	ARGs	n-Gp^c^	P+/G−^d^	P−/G+^e^	*r square*^f^	*P* value^g^
Broiler	Chl	105	*catA1*	17	92	4	0.018	0.067
Layer		85		10	77	2	0.026	**0.027**
Broiler		105	*cmlA*	49	66	10	0.107	**< 0.001**
Layer		85		35	54	4	0.169	**< 0.001**
Broiler	Ery	164	*ereA*	54	113	3	0.033	**0.009**
Layer		177		65	115	3	0.001	0.672
Broiler	Gen	55	*aac-3-IV*	50	11	6	0.610	**< 0.001**
Layer		50		44	7	1	**0.791**	**< 0.001**
Broiler	Tet	160	*tetA*	143	20	3	0.483	**< 0.001**
Layer		165		151	19	5	0.243	**< 0.001**
Broiler	Str	120	*aadA1*	66	56	2	0.275	**< 0.001**
Layer		150		66	91	7	0.023	**0.039**

^a^*Chl* Chloramphenicol, *Ery* Erythromycin, *Gen* Gentamicin, *Tet* Tetracycline, *Str* Streptomycin. Please note that the correlation between trimethoprim-sulfonamide and *sul1* was not included in this table because *sul1* is responsible for resistance to sulfamethoxazole only not to the trimethoprim-sulfonamide combination.

^b^n-Pr: number of strains expressing phenotype resistant to the indicated antimicrobial agent.

^c^n-Gp: number of strains carrying the indicated resistance gene.

^d^P+/G−: number of phenotypically resistance strains (P+) with no resistance genes (G−) for the antimicrobial identified.

^e^P−/G+: number of phenotypically susceptible strains (P−) with one or more resistance genes (G+) for antimicrobials.

^f^*r square* = 1 indicates positive correlation, *r square* = 0 indicates no correlation. The highest correlation obtained is highlighted in bold.

^g^*P* values < 0.05 are highlighted in bold.

## Discussion

Chicken meat is a potential source of multi-drug resistant ESBL-producing *E. coli* strains, which are responsible for serious human health concerns worldwide^32^. In this molecular study, we isolated *E. coli* from chicken meat and examined the existence of ARGs. The high prevalence of antibiotic resistant *E. coli* isolates in our findings indicates that the raw chicken meat from retail poultry shops could be contaminated with antimicrobial-resistant *E. coli*. This is alarming for developing countries like Bangladesh, where retail poultry shops hardly maintain proper hygienic condition during processing of chicken meat.

In the current study, the overall prevalence of *E. coli* in chicken meat was 63.5% whereas, 65.67% of broiler and 61.33% of layer meat swabs tested positive for *E. coli.* Other research groups detected high frequency of *E. coli* in poultry meat^33–35^. In contrast, Ranjbar et al*.*^36^, Moawad et al.^37^ and Younis et al.^38^ showed lower prevalence of *E. coli* in raw chicken meat. Another study in India found that 78% of broiler chicken meat specimens from retail shops were contaminated with *E. coli*^39^. Jakaria et al.^40^ reported that in Bangladesh, the prevalence rates of *E. coli* in layer, broiler, and indigenous chicken were 78.67%, 82% and 70%, respectively.

In this study, the disc diffusion method showed relatively higher frequency of antibiotic resistance and multidrug resistance among the investigated *E. coli* isolates than the genotypic analysis. This may be due to the possible protective role of the tested genes against multiple (often related) antimicrobial drugs that are not structurally or mechanistically related^41^. We found that more than 80% of the tested *E. coli* isolates were resistant to the common medically used antibiotics, such as ampicillin, erythromycin, and tetracycline. These findings were more or less similar to the findings of other researchers^42,43^. A similar study in Ethiopia showed that *E. coli* isolates from broiler chicken were resistant to tetracycline (90%), streptomycin (78%), ampicillin (60%) and highly sensitive to gentamicin (77%)^44^.

In this study, among the seven tested non-beta-lactam antibiotic resistance genes, the prevalence of tetracycline (*tetA*), sulphonamides (*sul1*), and streptomycin (*aadA1*) resistance genes were the highest (72.58%, 44.67% and 33.50%, respectively), followed by erythromycin (*ereA*) (31.23%) and the two chloramphenicol resistant genes *cmlA* (22.05%) and *catA1*(7.09%). Our findings indicate that chicken meat *E. coli* could be a reservoir of resistance genes, which may later become transferred to other common pathogens.

In a similar study, the prevalence rates of *tetA, aadA1* and *sul1*ARGs in *E. coli* isolated from Vietnam were found to be 81%, 81%, and 27.1%, respectively^24^, while their respective rates in Portugal were 41.1%, 70.6% and 23.5%^45^. Additionally, Moawad et al.^37^ reported that *E. coli* isolates from poultry meat were resistant to tetracycline (80.9%), streptomycin (61.9%) and trimethoprim/sulphamethoxazole (61.9%), which is higher than the prevalence rates reported in our present study. In Bangladesh, 37–100% of the poultry-derived *E. coli* strains were found resistant to chloramphenicol, tetracycline, streptomycin, erythromycin and penicillin, as reported by Rahman et al. and Islam et al.^46,47^. The lowest prevalence rate was observed with *aac-3-IV* (gentamicin resistance gene; 24.67%) which may be attributed to the very low absorption rate of gentamycin in poultry^48^.

We showed that 75.06% of our *E. coli* isolates were resistant to at least three antibiotics. In a study conducted in Iran, a high prevalence rate (64.91%) of MDR strains among *E. coli* isolates from commercial chicken meat has been reported^49^. Studies have demonstrated even higher prevalence rates of MDR *E. coli* in broiler (94%) and layer (60%) chicken in India^50^ and in Nepal (80.0%)^51^. Such high prevalence of MDR isolates may be due to misuse of antibiotics, which may ultimately replace the drug sensitive microorganisms in an antibiotic saturated environment^52^.

In our study, a total of 53 (13.91%) beta-lactam antibiotic resistant *E. coli* isolates were identified from broiler (*n* = 33; 16.75%) and layer (*n* = 20; 10.87%) chicken meat. Among the 5 types of *bla* genes (TEM, CTX-M, CTX-M-1, CTX-M-2 and SHV) tested by multiplex PCR in the current study, only the *bla*_SHV_ gene was detected in our *E. coli* isolates. In a study conducted in Netherlands, *bla*_CTX-M-1_(58.1%) has been found to be the most common gene in chicken meat, followed by *bla*_TEM-52_ (14%) and *bla*_SHV-12_ (14%)^53^. In another study conducted in Egypt, TEM, CTX-M and SHV genes have been detected in 57.55%, 46.23% and 23.58% of the isolates, respectively^54^. Highly prevalent ESBL-producing *E. coli* strains in meat samples from broiler (87%) and layer (42%) chickens have been previously reported in India^50^, Nepal (36.9%)^51^ and Vietnam (37%)^55^. In a previous study, the AmpC gene has been found to be mostly plasmid-associated whereas the chromosomal AmpC was found in a small percentage of *E. coli*^56^. In Vietnam and Italy, 84.2%^24^ and 11.2%^57^, respectively, of the chicken meat isolates carried plasmid-associated AmpC genes, which is higher than the prevalence among the isolates of the current study. Over expression of ESBL and AmpC beta-lactamases in gram-negative bacteria may reduce therapeutic options for treatment of their infections by providing resistance to most beta-lactam antibiotics^58^.

The strong correlation between the phenotypes and genotypes of AMR in bacteria may indicate that the resistance to these antibiotics is mainly attributed to the presence of certain AMR genes in their genome. Based on this, a number of strong correlations between the phenotypes and genotypes of AMR in *E. coli* were found in our study, such as between gentamicin and *aac-3-IV,* tetracycline and *tetA,* and streptomycin and *aadA1*, indicating that the resistance to some antimicrobials may be mediated, at least partially, by a single gene. This finding is more or less similar to the results of previous studies^59,60^.

On the other hand, interestingly, we found that some strains possessed resistance phenotypes but did not have the corresponding ARGs and vice versa. This finding is similar to the results reported by Rosengren et al.^60^. A possible explanation is that resistance phenotypes can be expressed upon the stimulation of many different genetic factors, and that each factor may present a unique epidemiological character^61,62^. Also, this may be due to the co-selection pressure of one antimicrobial class on another. It is known that the use of a particular antimicrobial agent can select for resistance not only to its own, but also act as potential co-selection marker for other antimicrobials agents. It means the use of single antimicrobial agent can lead to the selection and co-selection of multiple resistance phenotypes and ARGs^63^. It is reported that the high prevalence of MDR isolates as well as their persistence is governed by co-selection processes, even in the absence of antibiotic selection pressure^64^.

In the same context, in the current study we found comparatively large number of phenotypic erythromycin resistance *E. coli* strains than those carrying the *ereA* gene. This might be the result of the carriage of erythromycin resistance genes other than that included in our study. Thus, further detailed investigation is necessary to unveil the exact mechanism of AMR in *E. coli* isolates of broiler and layer chickens produced in Bangladesh.

There is very limited data on antibiotic use in chicken production in Bangladesh. In addition to the AMR genes that could be detected given the available resources, there may be other AMR genes that can be revealed in future studies. Further studies on the roles of TEM, SHV, and CTX genes will be important.

## Conclusion

Our study aimed primarily to characterize the antibiotic resistant *E. coli* isolates from commercial broiler and layer chicken meat samples in greater Sylhet division of Bangladesh. The correlations between the phenotypic and the genotypic susceptibility were also explored. Overall, the prevalence of *E. coli* was 63.5% (381/600) in these samples, from which 75.06% (286/381) of the isolates were MDR and 50.13% (191/381) contained 3 to 5 MDR genes. The isolates from raw chicken meat were highly resistant to ampicillin, erythromycin and tetracycline and 13.91% (53/381) of the isolates contained beta-lactamase (ESBL and AmpC) producing genes. This situation is alarming for Bangladesh, where facilities for health care, surveillance for antibiotics medication, and facilities to detect MDR and ESBL genes are underdeveloped. Our results highlight the need to develop novel antibiotic with potent activity against MDR and ESBL-producing bacteria. At the same time, promoting the rational use of antibiotics in livestock, as well as adopting safe food handling and proper cooking practices are crucial to reduce or eliminate the risk from pathogenic antibiotic resistance bacteria originating from raw foods.
